# Risk Factors for Small Airway Obstruction among Chinese Island Residents: A Case-Control Study

**DOI:** 10.1371/journal.pone.0068556

**Published:** 2013-07-18

**Authors:** Yu-sheng Chen, Xiao-qin Li, Hong-ru Li, Yan Miao, Shuang-qing Lian, Feng-feng Lu, Xiao-li Yu, Yun-hua Lin, Ting Liu

**Affiliations:** 1 Department of Respiratory Medicine, Fujian Provincial Hospital, Provincial Clinic College of Fujian Medical University, Fuzhou, Fujian Province, P.R. China; 2 Department of General Medicine, Fujian Provincial Hospital, Provincial Clinic College of Clinical Medicine of Fujian Medical University, Fuzhou, Fujian Province, P.R. China; 3 Department of Infectious Disease, Fujian Provincial Hospital, Fuzhou, Fujian Province, P.R. China; University of Montreal, Canada

## Abstract

**Background:**

We investigated the prevalence of and risk factors for small airway obstruction (SAO) among Chinese island residents to establish means to prevent and treat SAO.

**Methods:**

From October 17, 2011 to November 1, 2011, a total of 2,873 residents aged >20 years who lived on the Huangqi Peninsula of Fujian were recruited by random cluster sampling. They were asked to complete a Burden of Obstructive Lung Disease (BOLD) questionnaire and underwent physical examinations and lung function evaluations. SAO was defined as a forced expiratory flow at 50% of vital capacity, Vmax50%, of less than 70% of predicted. Risk factors for SAO were assessed from among demographic and anthropometric variables, blood chemistry results, and questionnaire response items.

**Results:**

A total of 216 (7.52%) Chinese island residents were identified as having SAO (95 males; 121 females). Their survey and test results were compared with 432 age and sex-matched healthy controls (192 males; 240 females) for SAO risk factors. Among numerous factors investigated, only diabetes mellitus (p = 0.039), smoking index (SI, p<0.001 for SI>600), second hand smoke (p = 0.002), and lack of regular exercise (p<0.001) were significant risk factors for SAO.

**Conclusions:**

The risk factors for SAO among Chinese island residents appeared to be similar to those among people who live in high-density urban environments and impoverished rural areas. Public health policies and medical practices directed toward improving respiratory health for island residents should be comparable to those used for urban and rural dwellers.

## Background

Bronchial asthma and chronic obstructive pulmonary disease (COPD) are two important diseases that have significant adverse impacts on human health. Both bronchial asthma and COPD are characterized by airflow obstruction and chronic airway inflammation. Chronic inflammation can affect the entire respiratory tract, including the central and small airways. Thus, early identification and prevention of COPD and bronchial asthma are crucial.

Both genetic and environmental factors are thought to contribute to asthma and COPD susceptibility. However, the results of numerous genome-wide association studies for susceptibility genes have been largely disappointing, probably due to the small effects of numerous genes whose products interact and the highly variable, complex genetic make-ups of different human populations. Likewise, there are numerous environmental factors that may contribute to these diseases. Yet, these too show widely varying results due to vastly different environmental conditions worldwide.

For example, the “hygiene hypothesis” for asthma susceptibility first proposed by Strachan in 1989 [1] has been found to have very different implications for asthma and allergy prevalence worldwide, as shown by the large International Study of Asthma and Allergies in Childhood (ISAAC) [2]. Similarly, the large international Burden of Obstructive Lung Disease (BOLD) study found widely varying incidence of COPD for reasons that could not be completely accounted for [3]. Among other implications from these large international studies, it seems reasonable that studies of these complex disorders should focus more on underlying, less complex physiological mechanisms that contribute to these diseases and those factors that contribute to these mechanisms among more well-defined human populations.

Chronic respiratory diseases, particularly COPD, are becoming an increasing public health concern in China due to its efforts to expand industrialization [4]. Previous epidemiological studies in China identified risk factors for COPD, such as smoking and solid fuel use [5] and socioeconomic status [6]. Yet, as in other countries in which these types of surveys have been conducted, differences in disease incidence and the contributing factors may differ between urban and rural areas [7]–[9]; although, a common factor appears to be air quality, either within the home or the environment in general.

In addition to chronic airflow obstruction and airways inflammation, a significant predisposing physiological component of both bronchial asthma and COPD is small airway obstruction (SAO) [10]. However, few large population studies have been undertaken that focused on predisposing factors for SAO. In addition, island residents represent a special population. An island environment provides good ventilation, favorable air quality, and high humidity. These should be favorable for reduced incidence of chronic airway diseases and provide a good basis for identifying those factors most critical for disease predisposition. Yet, few studies have investigated those factors associated with SAO within these populations.

Thus, for the present study, a total of 2,873 island residents aged >20 years were screened who lived in Moss Green Town of Huangqi Peninsula of Fujian. Risk factors associated with SAO were investigated within this population. Our findings may establish means to prevent and control SAO, COPD, and bronchial asthma among Chinese island residents and may be more broadly applied to other areas with different environmental conditions.

## Subjects and Methods

### Study Subjects

From October 17, 2011 to November 1, 2011, a total of 2,873 island residents aged >20 years were recruited in Moss Green Town of the Huangqi Peninsula of Fujian by random cluster sampling. Each person had lived on this island for more than 1 year. Each was asked to complete a questionnaire and undergo a physical examination and lung function evaluation. A total of 359 residents had incomplete information, which gave a loss rate to follow-up of 12.5%.

Inclusion criteria were normal heart function and lung function, no respiratory tract infection during the previous 4 weeks, and no history of other diseases. Subjects were excluded if they had respiratory diseases (including bronchial asthma and COPD), cardiovascular diseases, or diseases of other systems that were identified by questionnaire surveys and lung function tests.

This study was approved by our Institutional Review Board (IRB) and written informed consent was obtained from all study participants.

### Questionnaires

We used the questionnaire for the Burden of Obstructive Lung Disease (BOLD) [3] developed by the Global initiative for chronic Obstructive Lung Disease (GOLD). This questionnaire was modified based on the particular conditions relevant to China. Questionnaire items included age, gender, occupation, factors that could potentially affect lung function (active smoking, passive smoking, residence environment, others), respiratory symptoms, alcohol consumption, exercise, snoring, diabetes, and cardiovascular and cerebrovascular diseases. Subjects with any history of smoking were regarded as smokers, even if they had stopped smoking. A smoking index (SI) was calculated by: SI = number of cigarettes per day × years of smoking. Exercise was defined as exercising (including manual work) for longer than 30 min per day.

### Anthropometric and physiological measurements

Height (m), weight (kg), blood pressure (mmHg), waist circumference (WC; cm), and hip circumference (HC; cm) were measured. Prior to measuring body weight and height, subjects had fasted, emptied their bladders, and removed shoes and caps. For WC measurements, subjects were asked to stand straight with both feet apart (25–35 cm). WC measurements were made at the midpoint between the lower border of the rib cage and the iliac crest. HC was measured at the widest point around the greater trochanter. The body mass index BMI and WC to HC ratio were calculated. BMI  = weight (kg)/height (m)^2^.

For blood pressure determinations, a subject was asked to sit and relax for 5–10 min. While keeping the right elbow at the same level as the heart, the systolic pressure was defined when the first Korotkoff sound was heard and the diastolic pressure was defined when the fifth Korotkoff sound was heard. Blood pressure was determined twice with the right arm and the mean blood pressure was calculated.

### Lung function tests

Lung function was determined with a CHEST GRAPH lung function instrument (H1-101) according to criteria developed by the American Thoracic Society (ATS). Lung function was tested at least three times; the difference in each parameter between two tests was not greater than 5% and the variation in FEV1 and FVC was <200 ml. The maximum value was used.

The following parameters were determined: Forced Vital Capacity (FVC), Forced Expiratory Volume in 1 second (FEV1), FEV1/FVC ratios, 50% Forced Expiratory Flow (FEF50%), 75% Forced Expiratory Flow (FEF75%), and 25% Forced Expiratory Flow (FEF25%).

### Blood chemistries

Fasting venous blood (5 ml) was collected and serum was separated and stored for later use. Serum levels of glutamyl transferase, cholesterol (Chol), triglycerides (TG), high-density lipoprotein cholesterol (HDLC), low-density lipoprotein cholesterol (LDLC), blood glucose, creatinine (Cr), and blood urea nitrogen were determined using an automated biochemical analyzer (LX20; Beckman Coulter, USA). Serum C reactive protein (CRP) was determined by nephelometry using an automated protein analyzer (BN II; Siemens, Germany). These tests were done along with quality control in the Department of Laboratory Examination of Fujian Province-owned Hospital (a tertiary general hospital).

### Diagnosis of small airway obstruction (SAO)

SAO was defined as a forced expiratory flow at 50% of vital capacity, Vmax50%, of less than 70% of predicted [11]–[13].

### Statistical analysis

Results for continuous variables were summarized by medians (IQR: Q1, Q3) due to non-normal distributions and were compared between groups using Mann-Whitney U tests. Results for categorical variables were summarized as n (%) and compared between groups using Chi-square tests or Fisher's exact tests if any cell numbers were <5. Multivariate logistic regression analysis was used to identify risk factors that might be associated with small airway obstruction (SAO). Variables that were significantly different between SAO case and control groups from univariate analysis (P<0.05) were entered into multivariate logistic regression analysis. Results are given as odds ratios (OR) with the corresponding 95% confidence intervals (95%CI.). All statistical assessments were two-tailed and considered significant for P<0.05. Statistical analyses used SPSS 17.0 statistics software (SPSS, Inc., Chicago, IL, USA).

## Results

A total of 2,873 inhabitants of the island in Fujian were screened from Oct.17 to Nov. 1, 2011. Based on our selection criteria, 216 subjects (95 males and 121 females) had small airway obstruction (case group). For comparative purposes, we also randomly selected 432 controls from this population (192 males and 240 females) so that cases and controls were matched for age and sex at a 1:2 ratio. These subjects' mean age was 50.9 yrs (range: 27 to 85 yrs). As indicators of large airway obstruction, the median FVC value for cases was 2.80 (2.42, 3.26), which was significantly lower than the median FVC for controls of 2.97 (2.50, 3.48) (p = 0.007). The median FEV1 value for cases of 2.21 (1.90, 2.57) was also significantly lower than the median FEV1 value for controls of 2.58 (2.24, 3.04) (p<0.001). However, for both cases and controls, there was no evidence of large airway obstruction.

Table 1 shows the demographic characteristics, medical history, living habits, and laboratory test results for case and controls groups. Both groups had similar distributions for demographic variables and medical history (all P>0.05). Regarding living habits, the case group as compared to the healthy control group had a higher percentage of those with diabetes mellitus (8.7% vs. 3.9%), higher smoking index (SI) values, and higher percentages of those exposed to 2^nd^ hand smoke (53.&% vs. 41.0%) and snorers (52.8% vs. 43.3%) (All p<0.05). The case group also had a lower percentage who did regular exercise than controls (69.4% vs. 85%; p<0.05).

**Table 1:** Subjects' demographics, medical history, living habits, and laboratory test results.

Variable	Control group (n = 432)	Case group (n = 216)	p-value
Demographics			
Age, yrs	50.5 (42.0, 58.0)	50.5 (42.0, 59.0)	0.970
Sex			0.911
Males	192 (44.4%)	95 (44.0%)	
Females	240 (55.6%)	121 (56.0%)	
BMI, kg/m^2^	23.36 (21.4, 26.0)	22.9 (21.3, 25.5)	0.656
Waist circumference (WC)	78.5 (73.0, 85.0)	80.5 (74.0, 86.0)	0.103
High WC(>80 cm)	132 (30.6%)	88 (40.7%)	0.010*
Low WC ≤80 cm)	300 (69.4%)	128 (59.3%)	
Hip circumference (HC), cm	94.0 (90.0, 98.0)	94.0 (90.0, 97.0)	0.526
Education			0.680
No formal education	141 (32.7%)	69 (31.9%)	
≤6 years	165 (38.2%)	75 (34.7%)	
7–12 years	100 (23.1%)	59 (27.3%)	
>12 years	26 (6.0%)	13 (6.1%)	
Occupation			0.483
Management	10 (2.3%)	7 (3.2%)	
Farmer	56 (13.0%)	18 (8.3%)	
Homemaker	101 (23.4%)	46 (21.3%)	
Businessman	24 (5.6%)	15 (6.9%)	
Fishers	91 (21.1%)	51 (23.6%)	
Others	150 (34.7%)	79 (36.6%)	
Medical history			
Hypertension			0.804
Yes	80 (26.4%)	39 (25.3%)	
No	223 (73.6%)	115 (74.7%)	
Diabetes mellitus			0.016*
Yes	15 (3.9%)	17 (8.7%)	
No	373 (86.3%)	179 (82.9%)	
Cardiovascular disease			0.955
Yes	17 (7.6%)	8 (7.8%)	
No	207 (47.9%)	95 (44.0%)	
Hyperlipidemia			1.000
Yes	8 (4.9%)	3 (4.5%)	
No	155 (95.1%)	63 (29.2%)	
Living habits			
Smoking index (SI)			0.004*
No	341 (78.9%)	154 (71.3%)	
0<SI<200	22 (5.1%)	8 (3.7%)	
200≦SI<400	32 (7.4%)	5 (2.3%)	
400≦SI<600	15 (3.5%)	10 (4.6%)	
SI>600	22 (5.1%)	39 (18.1%)	
2^nd^ hand smoke			0.002*
Yes	177 (41.0%)	116 (53.7%)	
No	255 (59.0%)	100 (46.3%)	
Alcohol drinking			0.771
Yes	92 (20.4%)	42 (19.5%)	
No	343 (79.6%)	174 (80.6%)	
Snoring			0.022*
Yes	187 (43.3%)	114 (52.8%)	
No	245 (56.7%)	102 (47.2%)	
Exercise			<0.001*
Yes	367 (85.0%)	150 (69.4%)	
No	65 (15.0%)	66 (30.6%)	
House type			0.656
Brick	129 (29.9%)	66 (30.6%)	
Adobe	17 (3.9%)	13 (6.0%)	
Multi-story building	277 (64.1%)	132 (61.0%)	
Unknown	9 (2.1%)	5 (2.4%)	
Sunny house			1.000
Yes	349 (80.8%)	174 (80.6%)	
No	82 (19.0%)	41 (19.0%)	
Unknown	1 (0.2%)	1 (0.5%)	
Lighting in the house			0.575
Good	280 (64.8%)	143 (66.2%)	
Normal	143 (33.1%)	67 (31.0%)	
Poor	9 (2.1%)	5 (2.3%)	
Unknown	0 (0%)	1 (0.5%)	
Ventilation			0.657
Good	284 (65.7%)	143 (66.2%)	
Normal	140 (32.4%)	68 (31.5%)	
Poor	8 (1.9%)	4 (1.9%)	
Unknown	0 (0%)	1 (0.5%)	
Range hood used			0.954
Yes	159 (36.8%)	80 (37.0%)	
No	273 (63.2%)	136 (63.0%)	
Laboratory examination			
γ-GT			0.577
Low (≤60 IU/L)	405 (93.8%)	200 (92.6%)	
High (>60 IU/L)	27 (6.3%)	16 (7.4%)	
Triglycerides			0.342
Low (≤1.7 mmol/L)	366 (84.7%)	189 (87.5%)	
High (>1.7 mmol/L)	66 (15.3%)	27 (12.5%)	
Total cholesterol			0.015*
Low (≤5.7 mmol/L)	374 (86.6%)	171 (79.2%)	
High (>5.7 mmol/L)	58 (13.4%)	45 (20.8%)	
HDL- Cholesterol			0.320
Low (≤1 mmol/L)	329 (76.2%)	172 (79.6%)	
High (>1 mmol/L)	103 (23.8%)	44 (20.4%)	
LDL- Cholesterol			0.609
Low (≤3.6 mmol/L)	382 (88.4%)	188 (87.0%)	
High (>3.6 mmol/L )	50 (11.6%)	28 (13.0%)	
Glucose			0.720
Low (≤6.2 mg/dL)	384 (88.9%)	194 (89.8%)	
High (>6.2 mg/dL )	48 (11.1%)	22 (10.2%)	
Creatinine			1.000
Low (≤135 μmol/L)	429 (99.3%)	215 (99.5%)	
High (>135 μmol/L)	3 (0.7%)	1 (0.5%)	
Uric acid, unit			0.706
Low (≤430 μmol/L)	392 (90.7%)	194 (89.8%)	
High (>430 μmol/L)	40 (9.3%)	22 (10.2%)	
hsCRP			0.004*
Low (≤0.867 mg/dL)	298 (69%)	124 (57.4%)	
High (>0.867 mg/dL)	134 (31%)	92 (42.6%)	

For some categories, subjects' data were not available: 191for hypertension, 65 for diabetes mellitus, 321 for cardiovascular disease, and 491 for hyperlipidemia.Smoking index (SI) =  number of cigarettes per day × years of smoking.

Results for continuous variables are medians (IQR: Q1, Q3) and compared between groups by Mann-Whitney U tests. Results for categorical variables are n(%) and were compared between groups by Chi-square tests or Fisher's exact tests if any cell numbers were <5.

* indicates significantly different between case and control groups.

For laboratory test results, the case group had higher percentages of those with high levels of serum cholesterol (20.8% vs. 13.4%) and hsCRP (42.6% vs. 31%; both p<0.05). However, on more detailed analysis using continuous values, only serum total cholesterol was slightly higher in the case group (median = 4.96; IQR: 4.37, 5.60) compared to the control group (median = 4.72; IQR:4.05, 5.40; p = 0.002). The results for other laboratory tests were comparable between the case and control groups (all p>0.05).

Based on the results of univariate analysis shown in Table 1, those variables that were significantly different between case and control groups were entered into multivariate logistic regression analysis, including diabetes mellitus (yes vs. no), waist circumference (high vs. low), SI (None, 0<SI<200, 200≦SI<400, 400≦SI<600, SI>600), 2^nd^ hand smoke, snoring, exercise, total cholesterol (high vs. low), and hsCRP (high vs. low). As shown in Table 2, based on this analysis, an association with small airway obstruction was only significant for diabetes mellitus, SI, 2^nd^ hand smoke, and exercise.

**Table 2:** Multivariate logistic regression analysis for small airway obstruction risk factors.

Variables	OR	(95%CI.)	P-value
Diabetes mellitus			
Yes	2.258	(1.042, 4.890)	0.039*
No	Reference		
Waist circumference			
High	1.537	(1.023, 2.310)	0.039*
Low	Reference		
Smoking index (SI)			
SI>600	4.044	(2.136, 7.656)	<.001*
400≦SI<600	1.468	(0.599, 3.602)	0.402
200≦SI<400	0.386	(0.139, 1.072)	0.068
0<SI<200	0.808	(0.324, 2.017)	0.648
None	Reference		
2^nd^ hand smoke			
Yes	1.535	(1.060, 2.224)	0.023*
No	Reference		
Snoring			
Yes	1.240	(0.843, 1.823)	0.275
No	Reference		
Exercise			
Yes	0.310	(0.200, 0.482)	<0.001*
No	Reference		
Total cholesterol			
High (>5.7 mmol/L)	1.566	(0.954, 2.571)	0.076
Low (≤5.7 mmol/L)	Reference		
hsCRP			
High (>0.867 mg/dL)	1.207	(0.811, 1.797)	0.353
Low (≤0.867 mg/dL)	Reference		

Smoking index (SI)  =  number of cigarettes per day × years of smoking.

Results are odds ratios (OR) with corresponding 95% confidence intervals (95%CI).

* indicates significant association with small airway obstruction.

Table 3 shows the clinical symptoms recorded for the case and control groups. Comparing the cases and controls, there were significant differences for cough (65% vs. 54.6%; p<0.05) and sputum cruentum (53.3% vs. 44%; p<0.05).

**Table 3:** Clinical symptoms recorded for case and control groups.

Variable	Control group (n = 150)	Case groups (n = 77)	p-value
Cough			0.024*
Yes	14 (9.3%)	17 (22.1%)	
No	68 (45.3%)	27 (35.1%)	
Yes, but only during respiratory tract infection	68 (45.3%)	33 (42.9%)	
Sputum cruentum			0.007*
Yes	9 (6.0%)	15 (19.5%)	
No	84 (56.0%)	36 (46.8%)	
Yes, but only during respiratory tract infection	57 (38.0%)	26 (33.8%)	
Anhelation			0.094
Yes	10 (6.7%)	12 (15.6%)	
No	131 (87.3%)	60 (77.9%)	
Yes, but only during respiratory tract infection	9 (6.0%)	5 (6.5%)	
Stridor			0.799
Yes	1 (0.7%)	2 (2.6%)	
No	147 (98.0%)	74 (96.1%)	
Yes, but only during respiratory tract infection	2 (1.3%)	1 (1.3%)	

Results are n (%) and were compared between groups by Chi-square test or Fisher's exact test if any cell number was <5.

* indicates significantly different between case and control groups.

## Discussion

Both COPD and bronchial asthma are characterized by significant airflow obstruction, with evidence for obstruction in the larger airways. The major difference between these two diseases is that obstruction in COPD is irreversible, whereas in bronchial asthma this obstruction is reversible either spontaneously or with medication (e.g., bronchodilators). However, during the early stage of COPD, some parameters that reflect large airway function (FEV1 and maximal voluntary ventilation) are usually within normal ranges, although small airways (<2 mm in diameter) function is compromised. The same appears to be true for bronchial asthma. Thus, it is crucial to identify and treat small airway obstruction (SAO) as early as possible [10]. This may be particularly critical in China that is undergoing rapid industrial growth, as previous studies in China have shown significant differences in COPD incidence with respect to smoking and solid-fuel use [5], socioeconomic status [6], urban versus rural areas [8], and, perhaps most importantly, peoples' lack of recognition of airway diseases and their burdens on physical and economic well-being [7].

We chose an island population as this environment provides good ventilation, favorable air quality, and high humidity. These should be favorable for reduced incidence of chronic airway diseases and provide a good basis for identifying those factors most critical for disease predisposition. Further, there were no extremes in terms of socioeconomic variables among our study subjects, which could introduce confounding factors unrelated to physiological mechanisms associated with SAO.

Our results showed that smoking and second-hand smoke were significant risk factors for SAO (OR  = 4.044; p<0.001 and OR = 1.742; p 0.002, respectively). Small airways are anatomically characterized by numerous branches, a large cross-sectional area, and low resistance to air flow. Cigarette smoke contains a number of harmful substances, including naphthalene salts, nicotine, carbon monoxide, and others. Once inhaled, these substances can induce chronic inflammation of the airways, lungs, and pulmonary blood vessels [14]. Smoking has been confirmed as a pathogenic factor for COPD and smoking can cause airway obstruction (both large and small airways). Studies have shown that inflammatory mediators, such as PP2A, IL-17A, and IL-17F, are increased and may cause damage to the small airways [15]–[18]. Of note, large airway obstruction was not found among these subjects. Thus, emphasizing an early diagnosis of SAO and promoting smoking abstinence may be important strategies for reducing the incidence of large airway obstruction and COPD.

Although not statistically significant, our results showed that a large waist circumference (WC) was a marginal risk factor for SAO (OR = 1.439; p = 0.058). WC is an indicator of central obesity. Fat in the abdomen directly affects movement of the diaphragm and thorax compliance [19]–[22]. Reduced ventilation may cause peripheral alveoli collapse. Under these conditions, the small airways cannot open, which results in increased small airways resistance and reduced maximum mid-expiratory flow (MMEF) [23], [24]. However, BMI had no effect on lung function. This might be because body weight and BMI do not reflect fat distribution [25], whereas WC is closely related to fat distribution.

We also found that subjects with a lower exercise frequency had a higher incidence of SAO (OR = 0.311; p<0.001). This may be partly related to an increased systemic inflammatory response, especially in the small airways. There is evidence that exercise can reduce the serum levels of inflammatory cytokines. Serum IL-6 levels are reduced in COPD patients who exercise frequently, which is helpful for controlling disease progression [26].

In addition, exercise may increase the production of anti-inflammatory cytokines and reduce the production of pro-inflammatory cytokines, which has been found in patients with chronic heart failure and type 2 diabetes [23], [27], [28]. Our results also indicated that diabetes was a risk factor (OR = 2.258; p = 0.039), which might also have reflected the systemic inflammatory status of SAO patients in this study.

However, exercise reflects multiple factors. Subjects who are unaware of the benefits of exercise might not appreciate their health status. These subjects usually are smokers, obese, and have a large WC. The elastic fibers in the airway extend in a radial manner and connect to the elastic fibers in the peripheral alveoli to form a network structure. Thus, the size of a small airway outlet is directly affected by lung capacity. Exercise may strengthen smooth muscles and increase lung capacity and those people who frequently exercise have relatively large small airway diameters, which assure small airways opening. Thus, their incidence of SAO is at a low level. However, the effects of exercise of different extents and the mechanisms underlying exercise induced reductions in inflammation will need to be investigated in future studies.

Our results did not establish that cooking with woods and exposure to dust were risk factors for SAO. Both of these factors are related to the residence environment. Electricity and coal gas were used as fuels by more than 85% of the families in our study. In addition, houses on an island have good ventilation due to their proximity to the sea.

The major limitation of our study was that we only examined an island population. Thus, whether our findings can be extrapolated to more general conditions will require additional studies.

In summary, in our island population, SAO was associated with diabetes, smoking, second hand smoke, and exercise. When evaluating lung function, we should be aware of those patients with SAO. We should strongly advise these individuals to stop smoking, increase their exercise, and control inflammation if necessary, which may effectively reduce the incidence of SAO and ameliorate the progression of chronic lung diseases.
